# Progressive Collapse Safety Evaluation of Truss Structures Considering Material Plasticity

**DOI:** 10.3390/ma14185135

**Published:** 2021-09-07

**Authors:** Sheng-En Fang, Chen Wu, Xiao-Hua Zhang, Li-Sen Zhang, Zhi-Bin Wang, Qing-Yi Zeng

**Affiliations:** 1 School of Civil Engineering, Fuzhou University, Fuzhou 350108, China; 200527005@fzu.edu.cn (C.W.); cexhzhang@fzu.edu.cn (X.-H.Z.); N190520008@fzu.edu.cn (L.-S.Z.); wangzhibin@fzu.edu.cn (Z.-B.W.); 2 National & Local Joint Engineering Research Center for Seismic and Disaster Informatization of Civil Engineering, Fuzhou University, Fuzhou 350108, China; 3 Xiamen Jiahe Construction Co., Ltd., Xiamen 361022, China; wavelet2006@126.com

**Keywords:** truss structures, progressive collapse analysis, plastic importance coefficients, bearing capacity coefficients, material plasticity

## Abstract

Theoretical or numerical progressive collapse analysis is necessary for important civil structures in case of unforeseen accidents. However, currently, most analytical research is carried out under the assumption of material elasticity for problem simplification, leading to the deviation of analysis results from actual situations. On this account, a progressive collapse analysis procedure for truss structures is proposed, based on the assumption of elastoplastic materials. A plastic importance coefficient was defined to express the importance of truss members in the entire system. The plastic deformations of members were involved in the construction of local and global stiffness matrices. The conceptual removal of a member was adopted, and the impact of the member loss on the truss system was quantified by bearing capacity coefficients, which were subsequently used to calculate the plastic importance coefficients. The member failure occurred when its bearing capacity arrived at the ultimate value, instead of the elastic limit. The extra bearing capacity was embodied by additional virtual loads. The progressive collapse analysis was performed by iterations until the truss became a geometrically unstable system. After that, the critical progressive collapse path inside the truss system was found according to the failure sequence of the members. Lastly, the proposed method was verified against both analytical and experimental truss structures. The critical progressive collapse path of the experimental truss was found by the failure sequence of damaged members. The experimental observation agreed well with the corresponding analytical scenario, proving the method feasibility.

## 1. Introduction

In the real world, civil infrastructures might face progressive collapse risks due to design errors, construction bias and unpredictable events [1]. Unexpected factors such as seismic loads [2] and corrosion [3] may lead to a collapse accident. The progressive collapse of a structure often starts from the failure of components within a local region. Such failure might overspread the whole structure, eventually leading to the collapse event [4]. Therefore, how to avoid progressive collapse is an important issue in structural design, construction and operation. Some effective measures have been proposed in the relevant design codes [5,6]. The basic idea focuses on the effect of local failure on the whole structure, which forms the realm of progressive collapse analysis for checking the vulnerability or robustness of a structure [7,8]. Progressive collapse analysis can be performed within a qualitative or quantitative framework. A qualitative procedure highly relies on an engineers’ experience, and thus, it is not suitable for complex structures. A quantitative method attempts to find alternative load paths after one or some components fail [9], which is implemented in a numerical or analytical way [10]. The failure patterns and paths are of primary concern, and the importance of all components are calculated and ranked to seek the critical failure paths [11,12]. Then, structural optimization and component strengthening can be carried out to prevent the potential progressive collapse [13].

The importance of a component in a structure system can be represented by a quantitative coefficient [14], whose value embodies the risk of losing the component to the entire structure. The negative effect of the missing component is simulated by the so-called conceptual removal method. A component is removed from the structure every time, and the mechanical behavior of the residual structure is analyzed to evaluate any negative effect [15]. A greater importance coefficient implies a higher possibility of collapse risk, meaning the damage or failure of this component is more likely to cause a progressive collapse of the structure. In other words, this coefficient is directly related to structural vulnerability [16], or its antonym structural robustness [17]. However, the expression of importance coefficient is more intuitive than a vulnerability or robustness index at the component level [18]. The progressive collapse paths can be initially sought based on the sequence of importance coefficients of all components.

As is known, truss structures have been widely used in civil construction, e.g., cold-formed steel trusses are popularly adopted as the fundamental structure of portal frame structures for constructing light-weight buildings [18,19]. To date, the importance coefficient analysis of truss structures is usually established on the assumption of elastic materials for simplicity [20,21,22]. A modified elastic compensation approach can be used to determine the ultimate plastic load capacity of a collapsed structure [22]. For truss string structures, the numerical simulation results under certain elastic support stiffness might be close to the experimental collapse observations [23]. However, in actual collapse events some structural components will experience their plasticity states under large internal forces. On the other hand, plastic behaviors are very difficult to take into account in theoretical deduction due to the involvement of material nonlinearity. The analysis procedures are always performed with the help of finite element computation, which gives a solution with great convenience but cannot express the quantitative causality inside a progressive collapse process.

In view of this drawback, this work attempts to improve the existing method by fully considering the elastoplasticity properties of materials [20]. A plastic importance coefficient is proposed for truss members under axial internal forces. The material plasticity is embodied in local and global stiffness matrices, and the bearing capacity of a truss after removing a member is first deduced, for the subsequent calculation of the plastic importance coefficient. The progressive collapse path of the truss structure can be defined according to the failure sequence of the members.

## 2. Theoretical Assumptions and Failure Criterion

The proposed method focuses on truss structures. The scheme of ‘conceptual removal’ in the design code [5,6] is adopted for evaluating the importance of a removed truss member. External loads are applied to truss joints, and all members only undergo axial forces. Moreover, a truss structure with its members is assumed to meet the design of the elastic ultimate bearing capacity. In addition, instability effects are not considered for compressive members, whose failure is caused by the loss of material strengths. Lastly, the directions and distributions of external loads remain stable until the truss totally fails.

**Figure 1:**
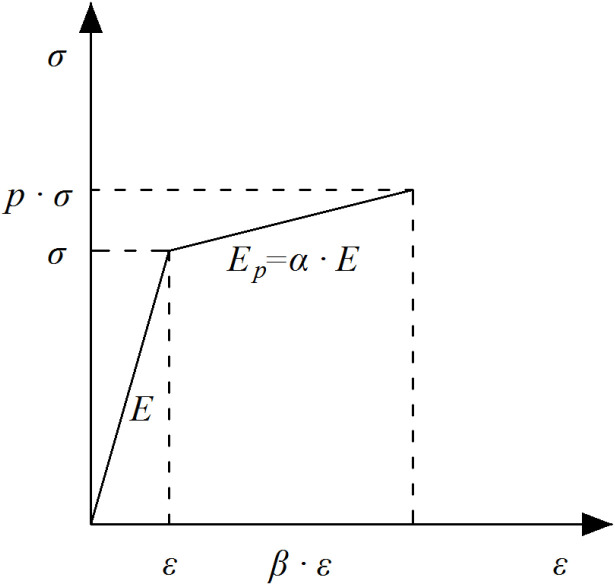
The constitutive model of materials.

Material plasticity is involved in establishing both local and global stiffness matrices of a truss structure. A bilinear elastoplastic constitutive model (Figure 1) is adopted to represent the material properties of truss members. The elastic moduli corresponding to the elastic and plastic stages are marked as *E* and $E_{p}=\alpha E$ respectively, where *α* gives the ratio of the tangent to elastic moduli. In the elastic deformation stage, the internal force of a member equals to its elastic stiffness *k* multiplying the elastic deformation $\Delta_{e}$. After that, the plastic deformation $\Delta_{p}$—which indicates when the damage happens—occurs when axial stress (strains) exceeds the elastic limit. When truss members enter their plastic stage, the axial stiffness decreases to $k_{p}=\alpha k$. The corresponding internal force is the elastic ultimate bearing capacity plus $k_{p}\cdot \Delta_{p}$. The member failure occurs when its internal stress reaches the strength limit. At that moment, the member stiffness decreases to zero with its internal force.

As to the failure (collapse) of a truss system, the static analysis terminates when the system becomes geometrically unstable. Under such a circumstance, the global stiffness matrix turns into a singular matrix.

## 3. Progressive Collapse Path of Truss Structures

Finding critical progressive collapse paths is the key for evaluating the anti-collapse performance of a structure. Numerical simulation is mostly employed for path searching [3,4,10]. However, it requires adequate modeling experience of engineers. On the other hand, analytical solutions are sometimes preferred by structural designers, who highly rely on design codes. After knowing the progressive collapse paths, engineers may set some alternative paths for load transferring during the design. Unfortunately, analytical methods are difficult to perform for real-world structures.

For practical usage, this study defines a new index of plastic importance coefficient to embody the importance of a member in a truss system under specific loading combinations. By adopting the conceptual removal approach [5,6], the effect of losing a member can be analyzed considering material plasticity, and the successive failures of the other members can be found by forming the critical collapse path.

### 3.1. Plastic Importance Coefficients

In [18], the importance coefficient $\eta_{i}$ of member *i* is defined as:

(1) $$\eta_{i}=\frac{\lambda_{0}-\lambda_{i}}{\lambda_{0}}$$

where λ denotes the ultimate bearing capacity coefficient of a truss structure at the elastic state; $\lambda_{0}$ denotes the coefficient of the undamaged structure; and $\lambda_{i}$ refers to the coefficient after removing member *i*.

It can be seen that λ gives the ratio of the ultimate bearing capacity of the current structure to its original load bearing capacity, and the solution of λ is the precondition for $\eta_{i}$. λ reflects the change in the load carrying performance of the structure before and after removing the member. Meanwhile, although $\eta_{i}$ corresponds to a member, its calculation is established on the mechanical capacity of the entire structure. Therefore, $\eta_{i}$ rationally reflects the contribution of a member to the bearing capacity of the truss system.

The original definition of $\eta_{i}$ has a clear physical meaning but cannot consider the plastic deformation of members, which departs from the actual situation in a collapse event. Hence, this study further proposes a plastic importance coefficient, $\eta_{i}^{p}$, that involves the plasticity properties of materials.

(2) $$\eta_{i}^{p}=\frac{\gamma \lambda_{0}-\lambda_{i}}{\gamma \lambda_{0}}$$

where $\lambda_{0}$ is redefined as the initial ultimate elastic bearing capacity coefficient of the undamaged structure; γ is an amplification factor to consider the effect of material plasticity on $\lambda_{0}$.

### 3.2. Bearing Capacity Coefficients

Suppose a load vector ***F*** is applied to a truss structure, the ultimate elastic deformations of all the members are given as follows:

(3) $$\Delta =AK_{0}^{-1}F$$

where **A** is the transformation matrix on the global displacement vector to members’ deformations; $K_{0}$ denotes the original stiffness matrix with its inverse $K_{0}^{-1}$.

After a truss member is removed from the original structure, the remaining members will gradually enter their plastic states until failure with the increase of external loads. Suppose at the *j*th load step, a number *x* of members (i=1,2,…,x) sequentially enter their plastic states. Among these members, non-failed members are marked as i=y,…,x (y≥1). The global stiffness loss δK of the truss structure is expressed by:

(4) $$\delta K=K_{0}-K_{j}=A^{T}({\tilde{K}}_{0}-{\tilde{K}}_{j})A=A^{T}\delta \tilde{K}A$$

where $K_{j}$ denote the stiffness matrix at the *j*th load step. The member-level matrices are similarly marked as ${\tilde{K}}_{0}$ and ${\tilde{K}}_{j}$ ($\delta \tilde{K}={\tilde{K}}_{0}-{\tilde{K}}_{j}$). Meanwhile, there is a relationship of $K\;=\;A^{T}\tilde{K}A$ among **A**, **K** and $\tilde{K}$. The corresponding deformation turns into:

(5) $$\Delta_{j}^{\left(1,2,\cdots ,x\right)}=AD^{\left(1,2,\cdots ,x\right)}=A{K_{j}}^{-1}(\lambda^{j-1}F+\delta F)$$

where $D^{\left(1,2,\cdots ,x\right)}$ is the global displacement vector at the *j*th load step; δF is the additional load caused by the members having the plastic deformations; and $\lambda^{j-1}$ is the ratio of the maximum element in **F** to the ultimate elastic bearing capacity at the *j*-1th load step. For the initial loading condition, $\lambda^{0}=\lambda_{0}=1$.

The ultimate bearing capacity coefficient after removing one or some members at each load step can be calculated using Equation (5). However, the solution process is very complex, and thus, Equation (5) is not suitable for practical applications. Due to this, this study develops a more efficient algorithm by transforming Equation (5) into:

(6) $$\begin{array}{ll} K_{j}D^{\left(1,2,\cdots ,x\right)} & =(K_{0}-\delta K)D^{\left(1,2,\cdots ,x\right)}\\ & =\lambda^{j-1}F+\delta F\\ & =\lambda^{j-1}F-A^{T}\left({\tilde{K}}_{0}^{\left(y,\cdots ,x\right)}-{\tilde{K}}_{j}^{\left(y,\cdots ,x\right)}\right)\Delta \end{array}$$

where ${\tilde{K}}_{0}^{\left(y,\cdots ,x\right)}$ equals to the operation on ${\tilde{K}}_{0}$ by defining all the elements (except i=y,…,x) as zero; ${\tilde{K}}_{j}^{\left(y,\cdots ,x\right)}$ comes from the same operation on $K_{j}$. Meanwhile, matrix **B** is defined as the transformation matrix between the constrained and unconstrained deformations of the members:

(7) $$B=A{\left(A^{T}\tilde{K}A\right)}^{-1}A^{T}\tilde{K}=AK^{-1}A^{T}\tilde{K}$$

After applying $AK_{0}^{-1}$ to both sides of Equation (6), one has:

(8) $$AK_{0}^{-1}\left(K_{0}-\delta K\right)D^{\left(1,2,\cdots ,x\right)}=AK_{0}^{-1}\left(\lambda^{j-1}F-A^{T}\left({\tilde{K}}_{0}^{\left(y,\cdots ,x\right)}-{\tilde{K}}_{j}^{\left(y,\cdots ,x\right)}\right)\Delta \right)$$

Introducing Equations (3), (4), and (7) into (8), one has:

(9) $$\begin{array}{ll} AK_{0}^{-1}\left(K_{0}-\delta K\right)D^{\left(1,2,\cdots ,x\right)} & =AD^{\left(1,2,\cdots ,x\right)}-AK_{0}^{-1}\delta KD^{\left(1,2,\cdots ,x\right)}\\ & =\Delta^{\left(1,2,\cdots ,x\right)}-AK_{0}^{-1}A^{T}\delta \tilde{K}AD^{\left(1,2,\cdots ,x\right)}\\ & =\left(I-AK_{0}^{-1}A^{T}{\tilde{K}}_{0}+AK_{0}^{-1}A^{T}{\tilde{K}}_{j}\right)\Delta^{\left(1,2,\cdots ,x\right)} \end{array}$$

Since $B_{0}=A{\left(A^{T}{\tilde{K}}_{0}A\right)}^{-1}A^{T}{\tilde{K}}_{0}=AK_{0}^{-1}A^{T}{\tilde{K}}_{0}$ and $B_{j}=A{\left(A^{T}{\tilde{K}}_{j}A\right)}^{-1}A^{T}{\tilde{K}}_{j}=AK_{j}^{-1}A^{T}{\tilde{K}}_{j}$, the expression of Equation (9) can be simplified as:

(10) $$AK_{0}^{-1}\left(K_{0}-\delta K\right)D^{\left(1,2,\cdots ,x\right)}=\left(I-B_{0}+B_{j}\right)\Delta^{\left(1,2,\cdots ,x\right)}$$

Considering the non-failed members (i=y,…,x), the left side of Equation (8) is further deduced as follows:

(11) $$AK_{0}^{-1}\left(\lambda^{j-1}F-A^{T}\left({\tilde{K}}_{0}^{\left(y,\cdots ,x\right)}-{\tilde{K}}_{j}^{\left(y,\cdots ,x\right)}\right)\Delta \right)=\lambda^{j-1}\Delta -\left(AK_{0}^{-1}A^{T}{\tilde{K}}_{0}^{\left(y,\cdots ,x\right)}-AK_{0}^{-1}A^{T}{\tilde{K}}_{j}^{\left(y,\cdots ,x\right)}\right)\Delta$$

Since $B_{0}^{\left(y,\cdots ,x\right)}=AK_{0}^{-1}A^{T}{\tilde{K}}_{0}^{\left(y,\cdots ,x\right)}$ and $B_{j}^{\left(y,\cdots ,x\right)}=AK_{j}^{-1}A^{T}{\tilde{K}}_{j}^{\left(y,\cdots ,x\right)}$, the expression of Equation (11) can also be simplified as

(12) $$AK_{0}^{-1}\left(\lambda^{j-1}F-A^{T}{\tilde{K}}_{0}^{\left(y,\cdots ,x\right)}\Delta \right)=\left(\lambda^{j-1}I-B_{0}^{\left(y,\cdots ,x\right)}+B_{j}^{\left(y,\cdots ,x\right)}\right)\Delta$$

Combining Equation (10) with (12), the axial deformation of each member is given as follows:

(13) $$\Delta^{\left(1,2,\cdots ,x\right)}={\left(I-B_{0}+B_{j}\right)}^{-1}\left(\lambda^{j-1}I-B_{0}^{\left(y,\cdots ,x\right)}+B_{j}^{\left(y,\cdots ,x\right)}\right)\Delta$$

Based on Equation (13), the member deformations can be calculated under the conditions of $\lambda^{j-1}$ and some plastic/failed members. Further, their proportion to the ultimate elastic limit deformation, $\Delta^{\left(1,2,\cdots ,x\right)}/\Delta$, can be obtained for estimating the importance coefficients.

### 3.3. Solution Procedure of Bearing Capacity Coefficient

Given an initial external load set of ***F***_1_ = {1} is applied to a truss structure with *n* members, one has $\lambda_{0}^{e}=1$. Meanwhile, the ratio of the ultimate plastic to elastic strain of member *i* is defined as $\beta_{i}$. If member 1 (*i* =1) first fails under ***F***_1_, this member is removed from the truss and its axial stiffness *k*_1_ is modified to zero in the global stiffness matrix. The remaining members constitute a new truss structure, and the progressive collapse analysis continues until the truss becomes a geometrically unstable system. The following paragraph demonstrates how to obtain $\lambda_{1}$ after removing member 1.

Suppose member 1 has been removed, the deformation $\Delta_{i}^{\vec{1}}$ of each member in the newly formed truss is first calculated under the initial load set of ***F***_1_. The superscript $\vec{1}$ means the removal of member 1. Then, the ratios of $\Delta_{i}^{\vec{1}}/\Delta_{i}$ (i≠1) are calculated and arranged by sequence according to their absolute values, from large to small. The sequence of all the remaining members is numbered as 2,3,…,n, indicating that member 2 will enter its plastic state first. The critical load set for the newly formed truss is $F_{2}$ ($F_{2}=\Delta_{2}/\Delta_{2}^{\vec{1}}\cdot F_{1}$), implying that when the external load increases from 0 to $F_{2}$, the plastic deformation will occur in member 2 with the axial stiffness of $k_{p,2}=\alpha k_{2}$. The next step is to judge whether member 2 fails based on the discriminant of $\Delta_{2}^{\vec{1}}/\Delta_{2}<\beta_{2}$. Once $\Delta_{2}^{\vec{1}}/\Delta_{2}\geq \beta_{2}$, member 2 fails. Simultaneously, the same judgment procedure is performed on the other members (i=3,…,n). $\Delta_{i}^{\vec{1}}/\Delta_{i}<1$ indicates a member stays at the elastic state, while $\Delta_{i}^{\vec{1}}/\Delta_{i}\geq 1$ implies the plastic state.

In the next iteration, member $x_{2}$ is removed on the condition of $F_{2}$. The ratio of $\Delta_{i}^{\vec{1},\vec{2}}/\Delta_{i}$ (i=3,4,…,n) is recalculated, and the current states of all the remaining members are determined. In the new global stiffness matrix, $k_{1}=k_{2}=0$ and $k_{p,3}^{}=\alpha k_{3}$. Then, the state judgment for all the remaining members is carried out again. The analytical iterations continue until the truss finally becomes a geometrically unstable system. At that moment, the ratio of the last to the initial external load is defined as $\lambda_{1}$.

It can be seen that during the solution process, some members are removed one by one until the truss fails. The removal sequence comprises a critical collapse path of the truss structure. Then, the identical solution procedure is repeated for $\lambda_{i}\left(i=2,3,\ldots ,n\right)$ to find other potential collapse paths. For example, when member 2 is removed from the truss structure, the solution iterations start looking for $\lambda_{2}$.

### 3.4. Features of Plastic Importance Coefficient

With $\lambda_{i}$ of all the members known, their $\eta_{i}^{p}$ can be calculated using Equation (2). The solution procedure of $\lambda_{i}$ shows that $\eta_{i}^{p}$ takes into account both elastic and plastic properties of materials. The state transformation of a member from elasticity to plasticity is reflected by the variation of external loads. Meanwhile, the structural topology is also under consideration because the geometrical stability of the truss structure determines its failure modes. By these means, the progressive collapse analysis is practically close to the real situation.

It is noted that if the instability effects caused by the initial imperfection of compressed members are considered in the analysis, the potential solution is to adjust the relevant parameters in the geometric stiffness matrices.

## 4. An Analytical Truss Structure

**Figure 2:**
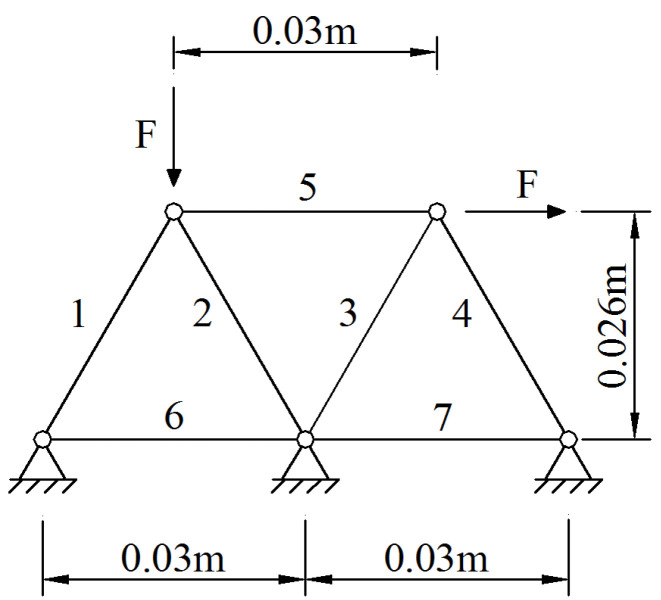
A plane truss structure (unit: m).

An analytical truss structure (Figure 2) under an initial external load $F_{1}=1$ kN was first adopted for validation. All the members had a length of 0.03 m and a sectional area of 9.2 mm^2^. Their elastic and tangent moduli were given as *E* = 190 GPa, and $E_{p}=\alpha E=0.0494\times 190=9.4$ GPa. The yield and ultimate strengths were assumed to be 360 MPa and 530 MPa, respectively. Thus, the elastic ultimate axial deformation was 0.057 mm, and the ratio of the plastic to elastic ultimate strain was $\beta_{i}=9.526$ for all members.

### 4.1. Plastic Importance Coefficient of Members

The amplification factor of the undamaged truss was first calculated with γ=5.5385, indicating that the original truss would fail under a load of $\gamma F_{1}=5.5385$ kN. Then, $\lambda_{i}$ (i=1,2,…,7) was analyzed by removing the members one by one. The solution process for $\lambda_{1}$ is given here as an example.

Step 1: After member 1 was removed, the deformation ratio of the new truss structure under $F_{1}=1$ kN was:

$$\Delta_{i}^{\vec{1}}/\Delta_{i}={\left\{\begin{array}{cccccc} 0.3475 & 0.1272 & 0.1272 & 0.1737 & 0 & 0 \end{array}\right\}}^{T}\left(i=2,3,\ldots ,7\right)$$

The elements in the vector $\Delta_{i}^{\vec{1}}/\Delta_{i}$ corresponded to the remaining members. For instance, 0.3475 belonged to member 2, and 0.1272 referred to member 3. Therefore, when the critical load factor was 1/0.3475=2.8778, member 2 was about to enter its plastic state.

Step 2: The global stiffness matrix of the new truss with six members was reconstructed, and the deformation ratio was recalculated under the load of $F_{2}=2.8788\times F_{1}=2.8788$ kN to become:

$$\Delta_{i}^{\vec{1}}/\Delta_{i}={\left\{\begin{array}{cccccc} 1.000 & 0.3660 & 0.3660 & 0.5000 & 0 & 0 \end{array}\right\}}^{T}$$

Therefore, once the external load increased up to $F_{2}/0.5000=2.8778/0.5000=5.7556$ kN, member 5 was about to enter its plastic state. At that moment, the plastic deformation had already occurred in member 2. It was further observed that $\Delta_{2}^{\vec{1}}/\Delta_{2}\geq \beta_{2}=9.526$ when *F* reached 4.0899 kN, indicating the failure of member 2. Unfortunately, the truss structure became geometrically unstable, which meant the collapse of the truss system was caused by the combined effects of the conceptual removal of member 1 and the failure of member 2. Hence, $\lambda_{1}=4.0899$.

Step 3: The plastic importance coefficient of member 1 was calculated as follows:

$$\eta_{1}^{p}=\frac{\gamma \lambda_{0}-\lambda_{1}}{\gamma \lambda_{0}}=\frac{5.5385\times 1-4.0899}{5.5385\times 1}=0.2616$$

By removing the next member and repeating the previous steps, $\eta_{i}^{p}$ of the other six members were also obtained and are listed in Table 1. It is observed that the three diagonal members (i=2,3,4) have identical importance for the truss structures under the external loads shown in Figure 2. The loss of each of them will lead to a higher safety risk for the other four members. This is because the original truss was a statically indeterminate structure, which became statically determinate after removing any load-carrying member (i=1,2,3,4,5). For a statically determinate structure, the internal force of a member only relies on the structural geometrical topology. Changes in member stiffness only affect the amounts of axial deformations. Hence, if one of members 2, 3 and 4 is removed, the failure load coefficient of the newly formed statically determinate truss is consistent. Therefore, members 2, 3 and 4 had the same importance in the truss system.

On the contrary, $\eta_{6}=\eta_{7}=0$ implies the unimportant roles of members 6 and 7 inside the system, which accords with the true situation because they are zero members.

### 4.2. Progressive Collapse Process of the Truss

As is aforementioned, the failure sequence of the truss members can be sought under the precondition of removing a specific member. If member 1 is removed, member 2 will enter its plastic state and then fail. After that, the truss structure becomes a geometrically unstable system, resulting in its collapse. The progressive collapse process is marked as $\vec{1}\to \tilde{2}\to \vec{2}\to \mathrm{system}\;\mathrm{failure}$, where $\tilde{2}$ and $\vec{2}$ represent the plastic and failure states of member 2. Furthermore, the collapse analysis results of removing the seven members are given in Table 2.

## 5. An Experimental Truss Structure

**Figure 3:**
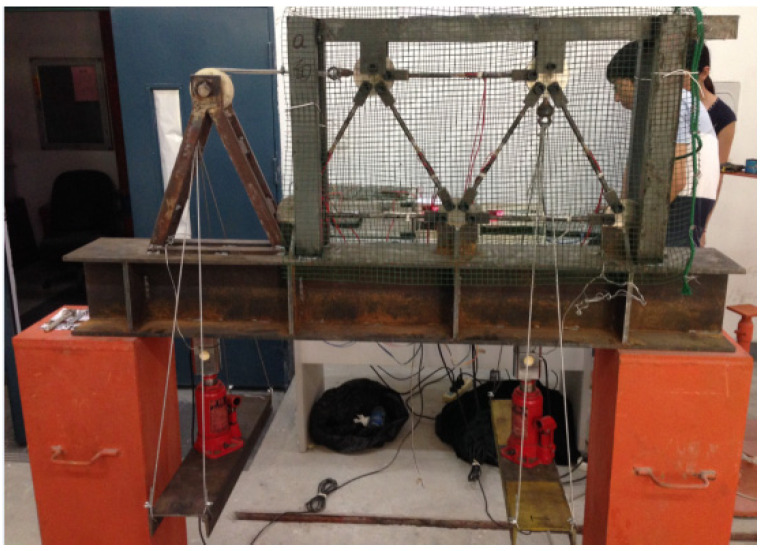
Progressive collapse experimental layout of the steel truss.

The progressive collapse experiment was performed on a steel truss structure shown in Figure 3. The truss was welded on an I-shape steel base. All the members were fabricated using the circular steel pipes with the nominal inner and outer diameters of 4 and 6 mm, respectively. The material properties of the steel were the same as those of the analytical truss in Section 4. The middle segments of the steel pipes were weakened with a thickness of 0.5 mm. The segment lengths were 30 mm where the strain gauges were mounted. The external forces were offered by two jacks and applied to two joints at the upper chords. The load increment was 0.2 kN in each setup, and the loads were held for 3 min until the strain measurements were stable. Therefore, the calculation schematic diagram of this truss was identical to that in Figure 2.

Meanwhile, to prevent the occurrence of buckling phenomenon, two steel sleeves were mounted on members 2 and 4 under compression. It is noted that for the experimental truss, the conceptual removal of members was replaced by direct loading on the structure in order to find the first damaged member, which also failed first as the jack load increased. This scheme did not violate the theoretical assumptions because the failed member no longer participated in the load carrying process, which could be regarded as performing “conceptual removal” on the truss system.

**Figure 4:**
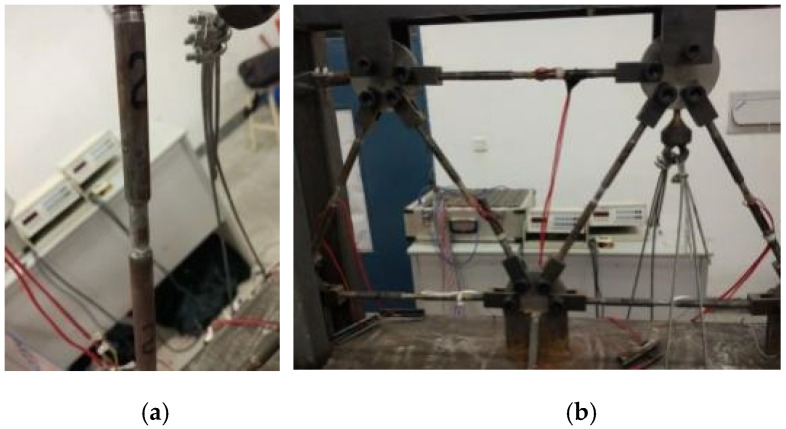
Failure modes of the truss members: (**a**) axial compressive failure of member 2; (**b**) tensile failure of member 3.

It was observed that the strain magnitudes of all the members did not exceed 2000 με until the jack loads reached 3.4 kN. All members stayed at the elastic state, and the measured strains of the zero members (6 and 7) were very small, indicating the rationality of the loading scheme. After that, member 2 first entered its plastic state, and then members 3 and 4 had plastic deformations. When the loads arrived at 4.0 kN, member 2 failed and a new truss was formed by the remaining six members (Figure 4a). When the loads increased up to 4.6 kN, member 3 broke under its ultimate internal tensile forces (Figure 4b). The newly formed truss with only five members became a geometrically unstable system resulting in the failure of the truss system. The strains inside members 2 and 3 both exceeded 20,000 με, and that of member 4 was greater than 14,000 με. At that moment, the magnitude of the strains of members 6 and 7 remained small. Hence, the experimental progressive collapse process of the truss structure was described as $\tilde{2}\to \tilde{3},\tilde{4}\to \vec{2}\to \vec{3},\tilde{4}\to \mathrm{system}\;\mathrm{failure}$. The second half of the process ($\vec{2}\to \vec{3},\tilde{4}\to \mathrm{system}\;\mathrm{failure}$) agreed well with the analytical observation (see scenario #2 in Table 2). Member 4 did not fail because the experimental truss could not have a “perfect” condition such as in the analytical model. However, this member was close to its failure state at that moment.

In conclusion, the experimental validation proved the feasibility of the proposed method in the progressive collapse analysis of a truss structure.

## 6. Conclusions

To investigate the progressive collapse performance of truss structures facing potential safety risks, this study proposed an analytical solution strategy based on the importance evaluation of members in a truss system, under the assumption of elastoplastic materials. The elastoplastic constitutive model, instead of the commonly used elastic model, was adopted in the analysis. The global stiffness loss due to the plastic deformations of the truss members was embodied in the structural stiffness matrices, and additional loads were employed to consider the load bearing capacity improvement of the truss in its plastic stage. Subsequently, the importance of each member was expressed by the proposed plastic importance coefficient, which was derived from the bearing capacity coefficients of the truss structure, before and after losing the corresponding member. The solution process was performed by iterations, until the truss became a geometrically unstable system. The experimental validation showed that the proposed method could well predict the collapse sequence of the truss members.

## Figures and Tables

**Table 1:** Plastic importance coefficients of truss members.

Member	1	2	3	4	5	6	7
$\lambda_{i}$	4.0899	2.9441	2.9441	2.9441	4.7227	5.5385	5.5385
$\eta_{i}^{p}$	0.2616	0.4594	0.4594	0.4594	0.1473	0	0

**Table 2:** Progressive collapse paths of the truss structure.

Removed Member	Failure Sequence
1	$\tilde{1}\to \tilde{2}\to \vec{2}\to \mathrm{system}\;\mathrm{failure}$
2	$\vec{2}\to \tilde{3},\tilde{4}\to \vec{3},\vec{4}\to \mathrm{system}\;\mathrm{failure}$
3	$\vec{3}\to \tilde{2}\to \vec{2}\to \mathrm{system}\;\mathrm{failure}$
4	$\vec{4}\to \tilde{2}\to \vec{2}\to \mathrm{system}\;\mathrm{failure}$
5	$\vec{5}\to \tilde{3},\tilde{4}\to \vec{3},\vec{4}\to \mathrm{system}\;\mathrm{failure}$
6	$\vec{6}\to \tilde{2}\to \tilde{3},\tilde{4}\to \vec{2}\to \vec{3},\vec{4}\to \mathrm{system}\;\mathrm{failure}$
7	$\vec{7}\to \tilde{2}\to \tilde{3},\tilde{4}\to \vec{2}\to \vec{3},\vec{4}\to \mathrm{system}\;\mathrm{failure}$

## Data Availability

Not applicable.
